# Comparison of Muscle Transcriptome between Pigs with Divergent Meat Quality Phenotypes Identifies Genes Related to Muscle Metabolism and Structure

**DOI:** 10.1371/journal.pone.0033763

**Published:** 2012-03-21

**Authors:** Marie Damon, Joanna Wyszynska-Koko, Annie Vincent, Frédéric Hérault, Bénédicte Lebret

**Affiliations:** 1 INRA, UMR1348 Physiologie, Environnement et Génétique pour l'Animal et les Systèmes d'Elevage (PEGASE), Saint Gilles, France; 2 Agrocampus Ouest, UMR1348 Physiologie, Environnement et Génétique pour l'Animal et les Systèmes d'Elevage (PEGASE), Rennes, France; American University in Cairo, Egypt

## Abstract

**Background:**

Meat quality depends on physiological processes taking place in muscle tissue, which could involve a large pattern of genes associated with both muscle structural and metabolic features. Understanding the biological phenomena underlying muscle phenotype at slaughter is necessary to uncover meat quality development. Therefore, a muscle transcriptome analysis was undertaken to compare gene expression profiles between two highly contrasted pig breeds, Large White (LW) and Basque (B), reared in two different housing systems themselves influencing meat quality. LW is the most predominant breed used in pig industry, which exhibits standard meat quality attributes. B is an indigenous breed with low lean meat and high fat contents, high meat quality characteristics, and is genetically distant from other European pig breeds.

**Methodology/Principal Findings:**

Transcriptome analysis undertaken using a custom 15 K microarray, highlighted 1233 genes differentially expressed between breeds (multiple-test adjusted P-value<0.05), out of which 635 were highly expressed in the B and 598 highly expressed in the LW pigs. No difference in gene expression was found between housing systems. Besides, expression level of 12 differentially expressed genes quantified by real-time RT-PCR validated microarray data. Functional annotation clustering emphasized four main clusters associated to transcriptome breed differences: metabolic processes, skeletal muscle structure and organization, extracellular matrix, lysosome, and proteolysis, thereby highlighting many genes involved in muscle physiology and meat quality development.

**Conclusions/Significance:**

Altogether, these results will contribute to a better understanding of muscle physiology and of the biological and molecular processes underlying meat quality. Besides, this study is a first step towards the identification of molecular markers of pork quality and the subsequent development of control tools.

## Introduction

Growing market demand for lean meat has directed pig breeding programs to obtain modern meat type of fattener [1]. Intense selection aiming at improving pork production efficiency through increased daily gain and carcass leanness has resulted in improved growth rate and feed conversion ratio as well as lean meat content and loin eye area, and decreased back fat thickness and carcass fat content [2]. However, some meat quality traits playing an important role in consumer acceptance of pork, like water holding capacity, colour, pH, intramuscular fat (imf) content and tenderness, were also affected [3]. Meat quality is complex and depends on the interactive effects of pig genotype, environmental conditions, pre-slaughter handling and slaughtering procedure [4]. Moreover, meat quality determination, as a result of physiological processes taking place in muscle could involve a large pattern of genes associated with both muscle structural and metabolic features. Ascertaining the transcriptome expression profiles differences between selected and non selected breeds which exhibit great differences for muscle meat quality traits, could be helpful to understand the biological processes underlying the development of meat quality.

For this purpose, the experiment was conducted to study gene expression profiles in *Longissimus lumborum* muscle (LM) of two contrasted pig breeds in terms of carcass fatness and meat quality, Large White (LW) and Basque (B). LW is the most predominant breed used in modern pig industry, with high lean meat productivity, low fat content and high daily gain, but with standard meat quality. By contrast, B is a local, indigenous breed with low lean meat and high fat contents, high meat quality characteristics, and which is genetically distant from other European pig breeds [5], [6]. Furthermore, the present transcriptome analysis is the first one undertaken on the high meat quality B breed, despite the increasing number of publications focusing on gene expression in relation with pork quality [7]–[11].

The aim of our study was to investigate the LM transcriptome profiles of LW (n = 20) and B (n = 20) pigs in relation to muscle traits and meat quality, and thereby clarify the biological events that result in the great phenotypic differences reported in literature between these two breeds [6], [12] and improve our general understanding of the determination of pork quality. These two breeds of pigs were reared either in alternative (A, indoor bedding and free access to an outdoor area; n = 10 per breed) or conventional (C, fully slatted floor; n = 10 per breed) housing systems, already demonstrated to influence some muscle and meat quality traits [13].

In order to get accurate information regarding gene expression profiles, the transcriptome analysis was undertaken using a new and specific pig muscle microarray, the 15 K Genmascqchip, in which 85% of the probes have been linked to a unique annotated sequence, and to 9169 unique genes [14]. Functional analysis of Gene Ontology (GO) biological process (BP) terms and functional annotation clustering were undertaken, to highlight main relevant biological networks and genes associated with muscle physiology and meat quality.

## Results

### Growth, body composition and LM characteristics

As shown in Table 1, B and LW pigs displayed large differences regarding growth, body composition, LM composition and biophysical traits, and sensory quality of meat. At the same live weight, B pigs were older (+85 days) than the LW due to their lower growth rate, and exhibited higher backfat thickness and percentage (+75%) and lower percentage of loin (−30%). Regarding LM composition, water content was slightly higher in the LW, whereas total protein content was similar between the two breeds. LM collagen content and glycolytic potential (GP) were higher, but intramuscular fat (imf) content was lower (−51%) in the LW compared with the B pigs. LW pigs also exhibited higher meat drip loss and shear force, and lower scores of tenderness, juiciness and flavour than the B. No significant effect of the housing system was found on growth and body composition, as well as on LM composition and biophysical traits. Tenderness score was lower for meat from A versus C housing system (4.0 *vs.* 4.4, P = 0.018) but juiciness and flavour scores did not differ (P>0.10) according to housing system.

**Table 1:** Differences between Basque (B, n = 20) and Large White (LW, n = 20) breeds for growth, body composition, and Longissimus muscle characteristics.

	B	LW	RSD^a^	P-value^b^
***Growth and body composition***				
Live weight at slaughter, kg	141.8	146.5	7.9	7.1E-02
Age at slaughter, d	315	230	21	<1E-04
Average daily gain (35–145 kg), g/d	522	746	92	<1E-04
Hot carcass weight, kg	115.4	117.0	6.7	4.6E-01
Backfat thickness, mm	45.8	23.7	6.0	<1E-04
Backfat, % of half carcass	14.8	8.2	1.6	<1E-04
Loin, % of half carcass	18	23.5	1.0	<1E-04
***Longissimus muscle composition***				
Water, %	71.6	73.6	1.10	<1E-04
Protein, %	23.2	23.1	0.74	9.6E-01
Collagen %	0.38	0.42	0.04	5.5E-03
Thermal solubility of collagen, % of total collagen	9.8	12.0	1.4	<1E-04
Intramuscular fat, %	3.99	2.03	0.98	<1E-04
Glycolytic potential, µmol lactate/g	139	161	15	<1E-04
***Biophysical traits of Longissimus muscle***				
Drip loss 1–3 d p.m., %	1.0	2.7	1.0	<1E-04
Shear force of cooked meat, N/cm^2^	22.2	32.1	4.7	<1E-04
***Sensory quality of meat (Longissimus)*** c				
Tenderness^d^	5.0	3.5	0.5	<1E-04
Juiciness	3.5	2.7	0.8	2.9E-03
Flavour	4.5	4.2	0.3	4.8E-03

a Residual Standard Deviation.

b P value of breed effect.

c Score between 0 to 10.

d A significant effect of housing system was found (A: 4.0 and C: 4.4, P = 1.8E-02).

### Transcriptomic analysis

**Figure 1:**
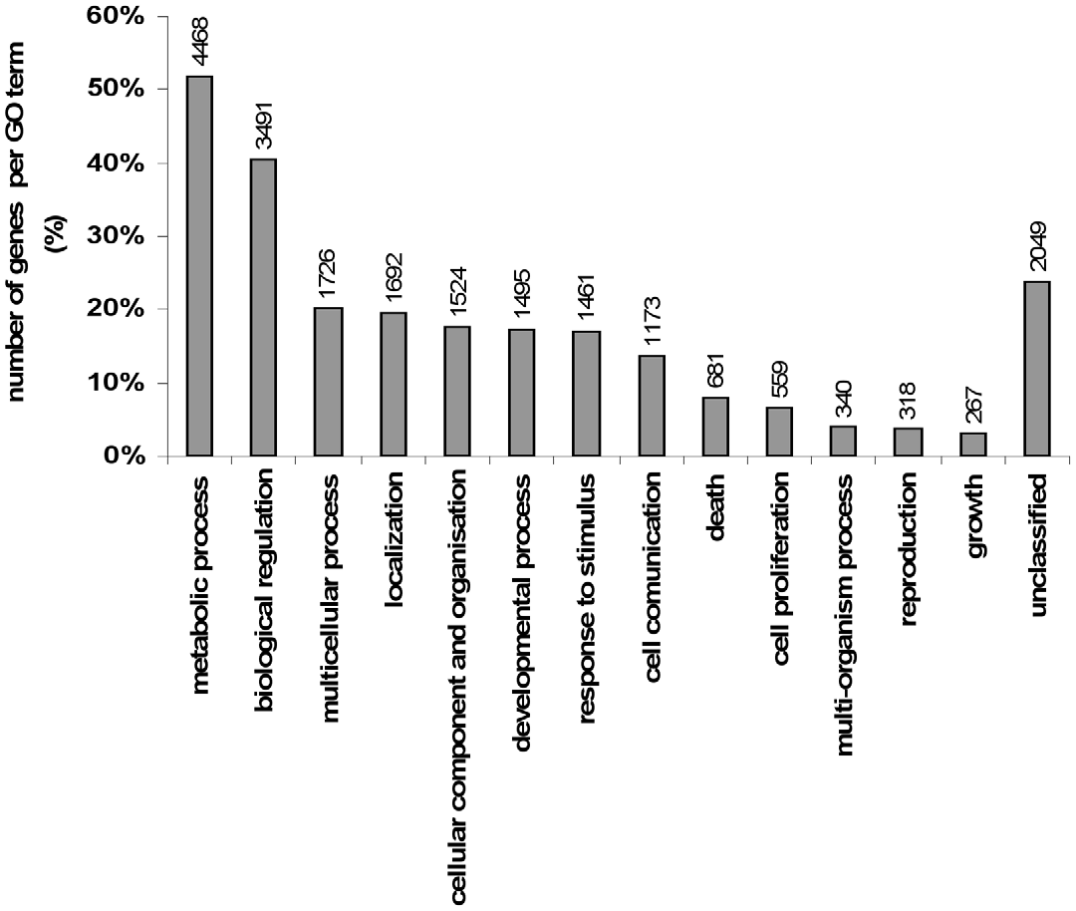
Microarray Biological Process (GO Slim) classification. Each Biological Process category is represented by a bar. The height of the bar represents the percentage of genes observed in the category. The number of genes per category is indicated upon the bars text.

Comparison of B and LW muscle transcriptome was achieved using a custom 15 K skeletal muscle pig microarray (Genmascqchip) [14] which is publicly available through Gene Expression Omnibus (GEO) [15] Platform accession no. GPL11016. Briefly, this new porcine skeletal muscle microarray is well annotated (more than 70%) and thereby allows studying a list of 9169 unique genes corresponding to 8622 human Entrez Gene ID. The WEB-based GEne SeT AnaLysis Toolkit [16] was used for the categorization of Gene Ontology (GO) terms for Biological Process (BP). The GO-slim (i.e. representing high-level GO) terms was used to focus on the most important processes. As shown in Figure 1, 13 biological processes were highlighted. The metabolic process category is the most important one (50% of the genes), whereas growth category accounts for less than 5% of the genes, and around 20% of the genes remained unclassified.

Muscle expression profiles of the two breeds (B, n = 20 and LW, n = 20) reared in the two different housing systems (C, n = 10 per breed and A, n = 10 per breed) were compared by transcriptomic analysis. LM genes expression was not modified according to the housing system, since no differentially expressed probe was found between A and C pigs. By contrast, we observed a strong breed effect on gene expression, with 12% of probes being differentially expressed between B and LW pigs (Benjamini-Hochberg (BH) adjusted P value<0.05). Genes showing a significant difference in expression between breeds were divided into 2 lists according to fold change (FC) value. Fold change value is expressed as the expression ratio of B to LW samples when genes are highly expressed in B pigs and as the expression ratio of LW to B samples when genes are highly expressed in LW pigs. The differentially expressed probes corresponded to 1233 unique annotated genes, out of which 635 were highly expressed in the B pigs (Table S1) whereas 598 genes were highly expressed in the LW pigs (Table S2). Full details of gene name, description, identification, FC and BH adjusted P value are reported in the Tables S1 and S2. In case of redundancy (i.e. more than one probe per gene), the FC were always similar within the probe set suggesting that microarray data were highly consistent. The most differentially expressed (FC>2) and well informative (i.e. with at least one associated GO BP term) genes are shown in Table 2 for genes highly expressed in the B pigs (2<FC≤2.6), and in Table 3 for genes highly expressed in the LW pigs (2<FC≤5). Among the 15 genes highly expressed in the LM of B pigs, four are involved in lipid metabolism: phospholipase A1 member A (*PLA1A*), protein farnesyltransferase subunit beta (*FNTB*), sphingomyelin phosphodiesterase acid-like 3A (*SMPDL3A*) and hormone-sensitive lipase (*LIPE*). Four genes are involved in transcription or translation: zinc finger protein 410 (*ZNF410*), zinc finger protein 24 (*ZNF24*), cytoplasmic polyadenylation element binding protein 2 (*CPEB2*) and keratin, type II cytoskeletal 7 (*KRT7*). Last, three genes are involved in ion transport or ion homeostasis: mitochondrial sodium/hydrogen exchanger NHA2 (*NHEDC2*), potassium large conductance calcium-activated channel (*KCNMA1*) and vacuolar fusion protein MON1 homolog A (*MON1A*). Among the 26 genes highly expressed in the LM of LW pigs, six are involved in transcription and RNA processing: zinc finger protein 7 (*ZNF7*), RNA polymerase-associated protein RTF1 (*RTF1*), interferon regulatory factor 8 (*IRF8*), LSM3 homolog (*LSM3*), sirtuin 3 (*SIRT3*) and ankyrin repeat domain 1 (*ANKRD1*). Five genes are involved in defence, immune system or stress: Interleukin-10 receptor subunit beta (*IL10RB*), glutathione peroxidase 8 (*GPX8*), asparagine synthetase (*ASNS*), Interferon regulatory factor 8 (*IRF8*) and *ANKRD1*. Four genes are involved in oxidation reduction: aldo-keto reductase family 1 member B1 (*AKR1B1*), *GPX8*, metaxin 3 (*MTX3*) and glyoxylate reductase/hydroxypyruvate reductase (*GRHPR*). Finally, three genes are involved in glucose metabolism: phosphoglucomutase 1 (*PGM1*), solute carrier family 5 member 4 (*SLC5A4*) and glyoxylate reductase/hydroxypyruvate reductase (*GRHPR*).

**Table 2:** Genes highly expressed in the Longissimus muscle of Basque pigs (n = 20).

Symbol^a^	Description	FC^b^	P-value^c^	Associated GO BP terms^d^
***FOS***	FBJ murine osteosarcoma viral oncogene	2.6	2.9E-02	Inflammatory response (6954), Response to oxidative stress(6979), Aging (7568), Learning (7612), Feeding behaviour (7631), Response to endogenous stimulus (9719), Response to extracellular stimulus (9991), Regulation of transcription (45449)
***CPEB2***	Cytoplasmic polyadenylation element binding protein 2	2.6	2.4E-06	Regulation of translation (6417)
***BVES***	Blood vessel epicardial substance	2.6	1.9E-07	Muscle organ development (7517)
***PLA1A***	Phospholipase A1 member A	2.6	3.1E-10	Lipid catabolic process (16042)
***ZNF410***	Zinc finger protein 410	2.4	2.4E-06	Transcription (6350)
***KRT7***	Keratin, type II cytoskeletal 7	2.3	4.1E-04	DNA replication (6260), Regulation of translation (6417), Cytoskeleton organization (7010), Cell cycle (7049)
***FNTB***	Protein farnesyltransferase subunit beta	2.3	9.2E-08	Response to wounding (9611), Regulation of cell proliferation (42127), Lipoprotein metabolic process (42157), Lipoprotein biosynthetic process (42158), Regulation of fibroblast proliferation (48145)
***NHEDC2***	Mitochondrial sodium/hydrogen exchanger NHA2	2.2	2.2E-03	Ion transport (6811)
***FBXO32***	F-box only protein 32	2.2	1.5E-04	Proteolysis (6508)
***KCNMA1***	Potassium large conductance calcium-activated channel, subfamily M, alpha member 1	2.2	1.5E-09	Cation homeostasis (55080), Response to hypoxia (1666), Muscle system process (3012), Circulatory system process (3013)
***SH3KBP1***	SH3-domain kinase binding protein 1	2.2	5.5E-04	Endocytosis (6897), Apoptosis (6915), Cell-cell signalling (7267)
***SMPDL3A***	Sphingomyelin phosphodiesterase, acid-like 3A	2.1	2.5E-02	Membrane lipid metabolic process (6643)
***LIPE***	Lipase, hormone-sensitive	2.1	1.1E-04	Protein amino acid phosphorylation (6468), Triglyceride metabolic process (6641)
***MON1A***	Vacuolar fusion protein MON1 homolog A	2.1	4.4E-10	Cellular ion homeostasis (6873), Protein secretion (9306)
***ZNF24***	Zinc finger protein 24	2.1	6.4E-11	Transcription (6350)

a Only genes with at least one associated GO BP term are presented in the Table.

b Fold Change value is expressed as the expression ratio of Basque to Large White samples.

c Benjamini and Hochberg adjusted P value.

d Gene Ontology identification numbers are shown in brackets.

**Table 3:** Genes highly expressed in the Longissimus muscle of Large White pigs (n = 20).

Symbol^a^	Description	FC^b^	P-value^c^	Associated GO BP terms^d^
***ZNF7***	Zinc finger protein 7	5.0	1.4E-03	Transcription (6350)
***PGM1***	Phosphoglucomutase 1	4.5	8.9E-08	Glucose metabolic process (6006)
***ADAMTS8***	ADAM metallopeptidase with thrombospondin type 1 motif, 8	4.3	2.0E-04	Proteolysis (6508), Regulation of cell proliferation (42127)
***LSM3***	LSM3 homolog	4.2	1.0E-07	RNA processing (6396)
***SPARC***	Secreted protein, acidic, cysteine-rich	2.9	2.3E-09	Skeletal system development (1501)
***DCTN3***	Dynactin subunit 3	2.8	4.3E-05	M phase of mitotic cell cycle (87)
***EBPL***	Emopamil binding protein-like	2.6	4.9E-09	Steroid metabolic process (8202)
***CLCN2***	Chloride channel protein 2	2.5	2.3E-09	Ion transport (6811)
***RRAS2***	Related RAS viral (r-ras) oncogene homolog 2	2.5	2.8E-06	Intracellular signaling cascade (7242), Regulation of cell migration (30334)
***RTF1***	RNA polymerase-associated protein RTF1 homolog	2.4	1.5E-02	Chromatin organization (6325), Transcription (6350)
***SLC5A4***	Solute carrier family 5, member 4	2.3	1.6E-06	Ion transport (6811), Carbohydrate transport (8643)
***CCRL2***	Chemokine (C-C motif) receptor-like 2	2.3	7.8E-07	Chemotaxis (6935)
***ANKRD1***	Ankyrin repeat domain 1	2.3	8.8E-03	Defense response (6952), Regulation of transcription (45449)
***SLC25A24***	Solute carrier family 25, member 24	2.3	2.3E-05	Transmembrane transport (55085)
***IRF8***	Interferon regulatory factor 8	2.2	2.3E-05	Immune system development (2520), Transcription (6350)
***SLC28A1***	Na/nucleoside cotransporter	2.2	2.7E-05	Nucleobase transport (15851)
***GRHPR***	Glyoxylate reductase/hydroxypyruvate reductase	2.2	4.0E-07	Cellular aldehyde metabolic process (6081), Secretion (46903), Oxidation reduction (55114)
***SIRT3***	Sirtuin 3	2.2	1.5E-18	Regulation of gene expression (40029)
***GINS2***	GINS complex subunit 2	2.2	8.3E-07	DNA replication (6260)
***ASNS***	Asparagine synthetase	2.2	1.1E-04	Regulation of mitotic cell cycle (7346), Cellular amino acid biosynthetic process (8652), Cellular response to starvation (9267), Response to endogenous stimulus (9719), Response to extracellular stimulus (9991), Regulation of cell death (10941), Cellular response to stress (33554)
***NEK3***	NIMA-related kinase 3	2.2	3.7E-08	Cell cycle (7049), Mitosis (7067)
***MTX3***	Metaxin 3	2.1	2.3E-09	Protein targeting to mitochondrion (6626)
***GPX8***	Glutathione peroxidase 8	2.1	2.1E-09	Response to oxidative stress (6979), Oxidation reduction (55114)
***AKR1B1***	Aldo-keto reductase family 1, member B1	2.1	9.6E-12	Oxidation reduction (55114)
***IL10RB***	Interleukin-10 receptor subunit beta	2.1	6.1E-06	Defense response (6952)
***RALB***	Ras-related protein Ral-B	2.1	2.0E-08	Intracellular signaling cascade (7242)

a Only genes with at least one associated GO BP term are presented in the Table.

b Fold Change value is expressed as the expression ratio of Large White to Basque samples.

c Benjamini and Hochberg adjusted P value.

d Gene Ontology identification numbers are shown in brackets.

### Validation of microarray analysis by quantitative RT-PCR

**Figure 2:**
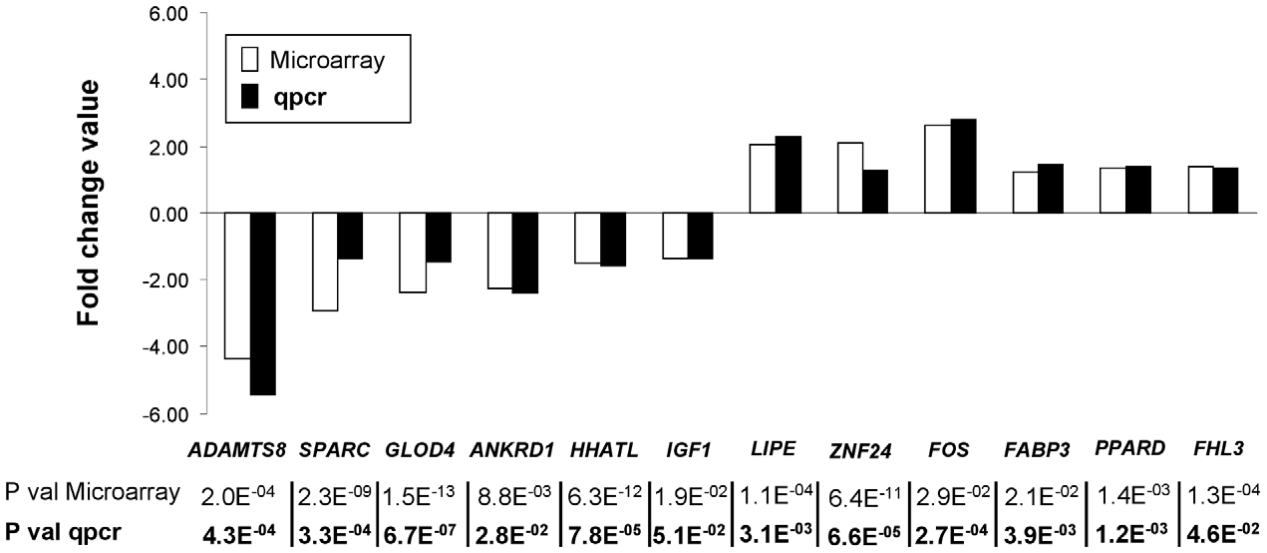
Validation of twelve microarray differentially expressed genes by quantitative PCR. Fold change value is expressed as the expression ratio of Basque (B, n = 20) to Large White (LW, n = 20) samples when genes are highly expressed in Basque pigs and as the negative expression ratio of LW to B samples when genes are highly expressed in LW pigs. Statistical significances are reported below the plot as Benjamini and Hochberg adjusted P value for microarray data and as Student t-test P value for qPCR data (bold case). *ADAMTS8*, ADAM metallopeptidase with thrombospondin type 1 motif, 8; *ANKRD1*, ankyrin repeat domain 1; *FABP3*, Fatty acid-binding protein, heart; *FHL3*, Four and a half LIM domains 3; *FOS*, FBJ murine osteosarcoma viral oncogene homolog; *GLOD4*, glyoxalase domain containing 4; *HHATL*, hedgehog acyltransferase-like; *IGF1*, insulin-like growth factor 1; *LIPE*, lipase, hormone-sensitive; *PPARD*, peroxisome proliferator-activated receptor delta; *SPARC*, secreted protein acidic and rich in cysteine; *ZNF24*, zinc finger protein 24.

Among the differentially expressed genes, twelve were chosen to validate the microarray differential expression results by real time quantitative PCR. Six genes highly expressed in the LW breed (*ADAMTS8*, *SPARC*, *GLOD4*, *ANKRD1*, *HHATL* and *IGF1*) and six genes highly expressed in the B breed (*LIPE*, *ZNF24*, *FOS*, *FABP3*, *PPARD* and *FHL3*) with FC extending in microarray analysis between 1.2 and 4.3, were thus analysed by RT-PCR. The results are shown in Figure 2. All these 12 genes were also found differentially expressed between the two breeds by RT-PCR methodology, and for each gene, the FC values were similar between the two methodologies used, i.e. microarray and RT-PCR.

### Functional analysis of differential expression between breeds

The two lists of genes, the 635 genes highly expressed in the B pigs and the 598 genes highly expressed in the LW pigs were submitted to an enrichment analysis for GO BP using the Database for Annotation, Visualization and Integrated Discovery (DAVID) bioinformatic resources [17]–[19]. Significant results (P value≤0.05) are presented in Table 4. GO BP terms related to lipid metabolism and transport, carbohydrate metabolism and transcription, were enriched in the B highly expressed genes list. GO BP terms for biological adhesion, protein polymerisation, chemotaxis and cytoskeleton organization, were enriched in the LW highly expressed genes list. To reduce the redundancy and study functionally related genes into a network format, a functional annotation clustering was performed using DAVID tools [17]. We used the three GO terms, BP, cellular component (CC) and molecular function (MF), BIOCARTA (http://www.biocarta.com) and Kyoto Encyclopedia of Genes and Genomes (KEGG) [20] pathways to build on biological modules consisting of clusters of related functional terms, for both B (Table 5) and LW (Table 6) highly expressed genes lists. An enrichment score of 1.3 which is equivalent to non-log scale P-value of 0.05, has been used as threshold for cluster significance according to Huang et al. [19]. Six enrichment groups were found to be significant with an enrichment score higher than 1.3, in the B highly expressed genes list. Three groups of genes were functionally categorized as genes of cytoskeleton (cluster 1-B), vacuole and lysosome (cluster 2-B) and glucose metabolic process (cluster 4-B). The three others clusters (clusters 3-B, 5-B and 6-B) were connected with transcription process. Regarding the LW highly expressed genes list, eight clusters related to extracellular region and collagen (clusters 1-LW, 2-LW and 5-LW), polysaccharide binding (cluster 3-LW), cell motion (cluster 4-LW), contractile fiber and actin polymerization (clusters 6-LW and 7-LW) and chemotaxis (cluster 8-LW) were identified.

**Table 4:** Relevant biological processes significantly enriched in the two lists of differentially expressed genes.

ID^a^	Name	n_G_ b	P-value^c^
**Basque highly expressed genes list**			
30518	Steroid hormone receptor signaling pathway	10	5.4E-03
30522	Intracellular receptor-mediated signalling pathway	11	7.0E-03
2761	Regulation of myeloid leukocyte differentiation	7	7.3E-03
19216	Regulation of lipid metabolic process	13	7.6E-03
45670	Regulation of osteoclast differentiation	5	1.7E-02
16042	Lipid catabolic process	14	1.9E-02
6006	Glucose metabolic process	16	2.1E-02
31328	Positive regulation of cellular biosynthetic process	40	2.3E-02
51173	Positive regulation of nitrogen compound metabolic process	39	2.4E-02
9891	Positive regulation of biosynthetic process	40	2.7E-02
19318	Hexose metabolic process	18	2.9E-02
45941	Positive regulation of transcription	34	2.9E-02
45449	Regulation of transcription	107	3.0E-02
48511	Rhythmic process	9	3.1E-02
15908	Fatty acid transport	5	3.4E-02
45935	Positive regulation of nucleotide, nucleic acid metabolic process	37	3.5E-02
10628	Positive regulation of gene expression	34	3.7E-02
46545	Development of primary female sexual characteristics	7	3.9E-02
46660	Female sex differentiation	7	3.9E-02
6357	Regulation of transcription from RNA polymerase II promoter	41	4.0E-02
10557	Positive regulation of macromolecule biosynthetic process	37	4.3E-02
9266	Response to temperature stimulus	8	4.4E-02
45637	Regulation of myeloid cell differentiation	8	4.4E-02
9266	Response to temperature stimulus	4	4.4E-02
45638	Negative regulation of myeloid cell differentiation	5	4.9E-02
**Large White highly expressed genes list**			
22610	Biological adhesion	37	1.4E-03
7155	Cell adhesion	37	1.4E-03
7517	Muscle organ development	21	1.9E-03
51258	Protein polymerization	6	8.1E-03
40012	Regulation of locomotion	16	8.6E-03
30334	Regulation of cell migration	15	1.2E-02
48232	Male gamete generation	17	1.3E-02
7283	Spermatogenesis	17	1.3E-02
51270	Regulation of cell motion	16	1.4E-02
6935	Chemotaxis	11	1.5E-02
42330	Taxis	11	1.5E-02
7015	Actin filament organization	9	1.8E-02
51674	Localization of cell	19	2.0E-02
48870	Cell motility	19	2.0E-02
6928	Cell motion	27	2.1E-02
16477	Cell migration	18	2.2E-02
15918	Sterol transport	5	2.6E-02
9261	Ribonucleotide catabolic process	5	2.6E-02
30301	Cholesterol transport	5	2.6E-02
48146	Positive regulation of fibroblast proliferation	5	3.2E-02
7276	Gamete generation	20	3.4E-02
30029	Actin filament-based process	19	3.8E-02
48729	Tissue morphogenesis	12	4.0E-02
8154	Actin polymerization or depolymerization	4	4.6E-02
2562	Somatic diversification of immune receptors via germline recombination	4	4.6E-02
6775	Fat-soluble vitamin metabolic process	4	4.6E-02
16444	Somatic cell DNA recombination	4	4.6E-02
30036	Actin cytoskeleton organization	18	4.8E-02

a Gene ontology identification number.

b n_G_ = number of genes in the category.

c Modified Fisher's exact test P value.

**Table 5:** Functional annotation clustering for genes highly expressed in the Longissimus muscle of Basque (B, n = 20) pigs.

Category	ID^a^	Name^b^	n_G_ c	P-value^d^
***Cluster 1-B enrichment score: 1.94***				
CC_FAT	5856	Cytoskeleton	70	5.8E-03
CC_FAT	43232	Intracellular non-membrane-bounded organelle	127	1.6E-02
CC_FAT	43228	Non-membrane-bounded organelle	127	1.6E-02
***Cluster 2-B enrichment score: 1.72***				
CC_FAT	5773	Vacuole	22	9.8E-03
CC_FAT	5764	Lysosome	18	2.6E-02
CC_FAT	323	Lytic vacuole	18	2.6E-02
***Cluster 3-B enrichment score: 1.55***				
MF_FAT	16563	Transcription activator activity	31	7.6E-03
MF_FAT	3713	Transcription coactivator activity	19	3.4E-02
MF_FAT	3712	Transcription cofactor activity	27	5.3E-02
MF_FAT	8134	Transcription factor binding	33	1.1E-01
***Cluster 4-B enrichment score: 1.51***				
BP_FAT	6006	Glucose metabolic process	16	1.7E-02
BP_FAT	19318	Hexose metabolic process	18	2.3E-02
BP_FAT	5996	Monosaccharide metabolic process	18	7.3E-02
***Cluster 5-B enrichment score: 1.46***				
BP_FAT	31328	Positive regulation of cellular biosynthetic process	40	1.6E-02
BP_FAT	51173	Positive regulation of nitrogen compound metabolic process	39	1.7E-02
BP_FAT	9891	Positive regulation of biosynthetic process	40	1.9E-02
BP_FAT	45941	Positive regulation of transcription	34	2.1E-02
BP_FAT	45935	Positive regulation of nucleobase, nucleoside, nucleotide and nucleic acid metabolic process	37	2.5E-02
BP_FAT	10628	Positive regulation of gene expression	34	2.7E-02
BP_FAT	6357	Regulation of transcription from RNA polymerase II promoter	41	2.8E-02
BP_FAT	10557	Positive regulation of macromolecule biosynthetic process	37	3.1E-02
BP_FAT	6355	Regulation of transcription, DNA-dependent	68	4.1E-02
BP_FAT	10604	Positive regulation of macromolecule metabolic process	47	5.9E-02
BP_FAT	45944	Positive regulation of transcription from RNA polymerase II promoter	22	6.1E-02
BP_FAT	51254	Positive regulation of RNA metabolic process	27	9.6E-02
BP_FAT	45893	Positive regulation of transcription, DNA-dependent	28	1.2E-01
***Cluster 6-B enrichment score: 1.37***				
MF_FAT	30528	Transcription regulator activity	76	2.5E-03
BP_FAT	51173	Positive regulation of nitrogen compound metabolic process	39	1.7E-02
BP_FAT	6357	Regulation of transcription from RNA polymerase II promoter	41	2.8E-02
BP_FAT	45449	Regulation of transcription	104	3.5E-02
BP_FAT	6355	Regulation of transcription, DNA-dependent	68	4.1E-02
BP_FAT	51252	Regulation of RNA metabolic process	69	5.7E-02
BP_FAT	6350	Transcription	82	8.3E-02
MF_FAT	3700	Transcription factor activity	39	9.1E-02
BP_FAT	51254	Positive regulation of RNA metabolic process	27	9.6E-02
MF_FAT	3677	DNA binding	85	1.1E-01
BP_FAT	45893	Positive regulation of transcription, DNA-dependent	26	1.2E-01

a Gene ontology identification number.

b Name of the ontology.

c n_G_, number of genes in the category.

d Modified Fisher's exact test P value.

**Table 6:** Functional annotation clustering for genes highly expressed in the Longissimus muscle of Large White (LW, n = 20) pigs.

Category	ID^a^	Name^b^	n_G_ c	P-value^d^
***Cluster 1-LW enrichment score: 6.01***				
CC_FAT	44421	Extracellular region part	57	8.1E-08
CC_FAT	5576	Extracellular region	82	8.6E-08
CC_FAT	5615	Extracellular space	36	1.3E-04
***Cluster 2-LW enrichment score: 4.87***				
CC_FAT	44421	Extracellular region part	57	8.1E-08
CC_FAT	5578	Proteinaceous extracellular matrix	27	1.5E-05
CC_FAT	31012	Extracellular matrix	28	2.2E-05
CC_FAT	44420	Extracellular matrix part	13	1.2E-03
***Cluster 3-LW enrichment score: 2.65***				
MF_FAT	30246	Carbohydrate binding	24	1.1E-04
MF_FAT	5539	Glycosaminoglycan binding	13	1.8E-03
MF_FAT	30247	Polysaccharide binding	13	3.4E-03
MF_FAT	1871	Pattern binding	13	3.4E-03
MF_FAT	8201	Heparin binding	9	2.4E-02
***Cluster 4-LW enrichment score: 1.8***				
BP_FAT	6928	Cell motion	27	1.5E-02
BP_FAT	51674	Localization of cell	19	1.5E-02
BP_FAT	48870	Cell motility	19	1.5E-02
BP_FAT	16477	Cell migration	18	1.8E-02
***Cluster 5-LW enrichment score: 1.49***				
CC_FAT	44420	Extracellular matrix part	13	1.2E-03
BP_FAT	30198	Extracellular matrix organization	9	6.0E-02
BP_FAT	43062	Extracellular structure organization	10	1.2E-01
BP_FAT	30199	Collagen fibril organization	4	1.3E-01
***Cluster 6-LW enrichment score: 1.39***				
CC_FAT	44449	Contractile fiber part	14	1.3E-02
CC_FAT	43292	Contractile fiber	14	2.3E-02
CC_FAT	30017	Sarcomere	12	2.7E-02
CC_FAT	30016	Myofibril	12	5.9E-02
CC_FAT	31674	I band	6	2.3E-01
***Cluster 7-LW enrichment score: 1.37***				
BP_FAT	51258	Protein polymerization	6	7.2E-03
BP_FAT	7015	Actin filament organization	9	1.6E-02
BP_FAT	8154	Actin polymerization or depolymerization	4	4.3E-02
BP_FAT	30041	Actin filament polymerization	3	5.7E-02
BP_FAT	43623	Cellular protein complex assembly	8	5.0E-01
***Cluster 8-LW enrichment score: 1.33***				
BP_FAT	6935	Chemotaxis	11	1.2E-02
BP_FAT	42330	Taxis	11	1.2E-02
BP_FAT	7626	Locomotory behavior	12	1.1E-01
BP_FAT	7610	Behavior	15	2.9E-01

a Gene ontology identification number.

b Name of the ontology.

c n_G_, number of genes in the category.

d modified Fisher's exact test P value.

## Discussion

Regarding genetic background, the B pig is an indigenous breed characterized as “unique” among 11 breeds belonging to seven European countries [5]. Despite an increasing number of publications focusing on gene expression in relation with pork quality [11], the present study is the first transcriptome analysis of this non-selected and high meat quality pig breed. Our objective was to clarify the biological events which could enlighten the muscle phenotypic differences reported in the literature between the B and LW pigs [6]. Transcriptional profiling of whole skeletal muscle tissue presents a challenge since changes in gene expression may reflect mRNA composition between various cell types existing in this tissue. However, even if we cannot ascribe expression changes to one specific cellular type we assume that myofiber is the major one and that comparison between breeds in the tissue as a whole is informative. After transcriptome analysis, 12 differentially-expressed genes between LW and B breeds have been properly validated by quantitative PCR analyses, thus demonstrating that the new GenmascqChip [14] is a powerful tool to study pig gene expression and thus get a better understanding of muscle physiology.

Even if the number of studies comparing gene expression of skeletal muscle in different pig genetic backgrounds is rather scarce [10], [21]–[22], the number of genes found differentially expressed between the two breeds in this study is in the same order of magnitude as found in literature. The high discrepancy in gene expression between B and LW pigs was rather balanced, with 635 and 598 genes highly expressed in B and in LW, respectively. This was associated with strong breed differences regarding growth performance, skeletal muscle characteristics and meat quality traits. In particular, a lower lean and a noticeable higher fat development were observed in B compared with LW, in agreement with previous B and LW comparisons on growth, carcass and muscle traits, and fat tissue metabolism [6], [12]. In order to slaughter the pigs from the two breeds at the same time and body weight, B pigs were put on experiment two months earlier and were three months older than LW pigs at slaughter because of their slower growth rate. Moreover, pigs from both breeds received the same amount of feed at a given live weight whereas the potential growth rate and appetite are much higher in LW than in B pigs. Thus, breed, age and feeding effects are confounded.

On the contrary, no difference in LM gene expression profiling was highlighted between A and C housing systems. Accordingly, LM composition, biophysical traits and meat quality were not affected by the housing system, except tenderness. This differs from a previous comparative study [13], thereby confirming that the animal response to husbandry varies according to genotype, environmental (climatic) conditions, etc. [23]. Moreover, differences for tenderness score were much lower between housing systems than between breeds. This supports the generally higher effect of genotype, especially for highly contrasted breeds, than housing conditions on muscle and meat traits [24]. However, we can not exclude that slaughtering conditions could have masked a potential housing effect established before slaughter.

A functional analysis of differential gene expression between LW and B pigs highlighted four main relevant biological networks associated to these breed differences: 1/metabolic processes, 2/cytoskeleton and contractile fiber, 3/extracellular matrix, and 4/vacuole, lysosome and proteolysis. Some examples of genes belonging to each of these categories will be discussed in relation to muscle physiology and meat quality.

### Metabolic processes

Enrichment analysis reveals that genes related to lipid metabolism process and fatty acid (FA) transport are more expressed in B than in LW breed. The higher *FABP3* (muscle fatty acid binding protein) expression and imf content of the B pigs corroborate several studies indicating this gene as a candidate for the control of imf deposition in pigs [25], [26]. In the B pigs, the higher expression of *ACACB* (acetyl-CoA carboxylase beta) considered as the rate limiting step in FA synthesis, agrees with Alfonso et al. [6] who reported a higher activity of acetyl-CoA carboxylase (ACC) in the muscle of B compared with LW. However, even if the B pigs seemed to deposit more imf than the LW, they also use more lipids as fuel substrates, and rely on fat oxidation and lipolysis to sustain their metabolic requirements. Indeed, *PPARD* (peroxisome proliferator-activated receptor delta), *SLC25A20* (carnitine/acylcarnitine translocase, which mediates the transport of acylcarnitines into the mitochondrial matrix for their oxidation) and *ETFDH* (electron-transferring-flavoprotein dehydrogenase), all related to the mitochondrial oxidation of FA, were more expressed in B than LW pigs. Regarding lipolysis, *PPAP2A* (phosphatidic acid phosphatase type 2A) and *LIPE* were found in the enriched functional category from the lipid catabolic process in the B highly expressed gene list. *PPAP2A* would play an active role in the hydrolysis and uptake of lipids from extracellular space [27], and a higher expression of *LIPE* in the “fatty” Jinhua than in the leaner Landrace breed has been observed [28]. Altogether, this indicates that a higher FA turn-over (including transport, synthesis and catabolism) could explain the breed discrepancy for imf content. However, since investigation has been conducted on the whole muscle tissue, we cannot exclude that a contribution of a higher number of adipocytes in B pigs could have mediated gene expression variations between the two breeds. Finally, the lower *SPARC* (secreted protein, acidic, cysteine-rich) expression in the LM of the B pigs is consistent with their higher imf and suggests a role of this gene in controlling imf content. Indeed, *SPARC* has been reported to inhibit adipogenesis and *SPARC*-null mice have been found to exhibit significantly more fat accumulation than wild-type mice [29].

Both functional annotation clustering (cluster 4-B) and enrichment analysis showed that glucose metabolism process is also of great importance in LM traits of B pigs. In this cluster, *AGL* (glycogen debranching enzyme) and *PHKB* (phosphorylase kinase beta) are responsible for the complete degradation of glycogen [30], [31]. This might suggest that B pigs would use glycogen as a muscle metabolic substrate whereas the LW would spare more glycogen, thus explaining their higher muscle GP. However, *PGM1* (phosphoglucomutase 1), *GAPDH* (glyceraldehyde 3 phosphate dehydrogenase) and *LDHA* (lactate dehydrogenase A) are up-regulated in the LW, indicating that these pigs would also rely on glucose to fulfil their energy requirements. The higher gene expression of glycolytic pathways in the LW agrees with the more glycolytic and less oxidative muscle metabolism generally observed in domestic compared to wild pigs [32].

Last, creatine kinase (*CK*) is an essential enzyme to maintain the ATP/ADP ratio in muscle cells and adjust energy availability for contraction. The higher expression of *CKB* (creatine kinase B chain; cytosolic) in the LW and of *CKMT2* (sarcomeric mitochondrial creatine kinase) in the B, suggest a rapid glycolytic ATP production during contraction in LW, while in B the mitochondrial ATP production would be transferred to myofiber via *CKMT2*, thereby reflecting a more oxidative muscle metabolism. Moreover, in agreement with the suggested cytosolic *CK* as a candidate protein marker for pork drip loss [33], *CKB* is more expressed in the LW which exhibited higher drip loss than the B pigs. Accordingly, cytosolic *CK* protein content was shown to be positively associated with meat lightness, which increases with drip [34].

### Cytoskeleton and contractile fiber

Three clusters, cluster 1-B (cytoskeleton) issued from B, and clusters 6-LW (contractile fiber) and 7-LW (actin filament organization) issued from LW functional annotation clustering analyses, reveal the skeletal muscle organization and structure as important features to characterize the breed differences in gene expression profiles.

The actin cytoskeleton is involved in many cellular processes [35], but the relationships between actin dynamics, cytoskeletal organization and muscle development are still unclear. Interestingly, *ABRA* (actin-binding Rho activating protein, also called *STARS*, striated muscle activator of Rho signaling) is highly expressed in the LM of B pigs. *ABRA* activates the serum response factor and leads to enhanced gene expression in skeletal muscle [36] and could thus contribute to the up-regulation of transcription found in the B pigs (clusters 5-B and 6-B). In the same way, *FOS*, the most differentially expressed gene in the B muscle, is a transcription factor known to induce myogenesis. Thus, *ABRA* and *FOS* are probably associated to the higher transcriptional activity observed in the B breed (clusters 3-B, 5-B and 6-B). Apart from transcription, *ABRA* is involved in skeletal muscle atrophy and hypertrophy [37]. In this cytoskeleton cluster, we also found *ABLIM2* (actin binding LIM protein family, member 2) recently identified as an *ABRA* interacting partner [38]. We can thus hypothesize that *ABRA* and *ABLIM2* could control the development of B muscle and maintain its cytoskeletal integrity. *LMOD2* (leiomodin 2) which interacts with actin filaments to promote thin filament elongation and probably their length [39] displays a higher expression in B pigs. This might have led to longer sarcomeres and thereby contributed to improve meat tenderness in this breed, since sarcomere length is positively associated with pork tenderness [40]. Furthermore, because muscles with short sarcomeres generally exhibit high drip loss [41], the higher expression of *LMOD2* could also be related to the lower drip loss of the B breed. Besides, *MYOZ1* (myozenin 1) also called calsarcin 2, is a sarcomeric calcineurin binding protein specific of striated muscles [42]. Calcineurin mediates calcium signalling and plays a central role in the regeneration and regulation of hypertrophy of skeletal muscle [43]. Calcineurin activity would be inhibited by *MYOZ1*, as shown in *MYOZ1* knock-out mice [42]. Therefore, we hypothesize that the higher expression of *MYOZ1* in the B muscle relates to their lower muscle mass through a reduced calcineurin activity, compared with the LW muscle. Similarly, *TRIM63* (tripartite motif containing 63, also called *MURF1*: muscle specific RING-finger protein-1) localized at both M- and Z-lines of the sarcomeres, has been related to muscle atrophy by gene expression profiling and knock-out studies [44]. This would suggest higher muscle atrophy in the B than in the LW, which could be related to their older age at slaughter and might contribute to their lower loin percentage. Finally, in this cytoskeleton cluster, *ZYX* (zyxin), a protein involved in stress fiber repair and maintenance of cytoskeleton integrity [45] was also found as highly expressed in the B pigs. However, we did not report any positive relationship between *ZYX* expression and meat drip loss in our study, contrarily to Ponsuksili et al. [8]. This may be explained by different experimental designs, i.e. contrasted breeds in the present work *versus* extreme drip loss groups within a F2 population in the study of Ponsuksili et al. [8], and indicates that the relationships between *ZYX* expression and pork quality remain further studies.

Apart from cytoskeleton, our results emphasise the importance of myofibrillar network and especially the contractile fiber (cluster 6-LW) in the muscle expression profile differences between B and LW. Major constituents of sarcomeres: *ACTA1* (actin alpha 1), *ACTA2* (actin alpha 2), *MYH1* (myosin heavy chain 1, IIx), *MYH3* (myosin heavy chain 3), *TPM1* (tropomyosin 1) and *TPM3* (tropomyosin 3) are all highly expressed in the LW, indicating that in this breed, LM is a fast skeletal muscle expressing IIx myosin. In this cluster, *NEB* (nebulin), which is abundantly expressed in skeletal muscle, plays a key role in thin filament length regulation, intermyofibrillar connectivity and calcium homeostasis [46]. Moreover, in Hanwoo cattle, *NEB* expression is associated with low marbling and high shear force [47], in accordance with the higher *NEB* expression in LW than B pigs, and their lower imf content and higher shear force value. The ANKRD1 (cardiac ankyrin repeat domain 1, also known as CARP) which belongs to this cluster, interacts with titin and other sarcomeric proteins to maintain sarcomeric integrity, and its expression is altered in several conditions such as exercise, muscle wasting, dystrophies and stress response [48]. In heart, *ANKRD1* interacts with protein *CASQ2* (calsequestrin 2) which stores Ca^2+^ inside the sarcoplasmic reticulum and modulates Ca^2+^ homeostasis [49]. Thus, *ANKRD1* seems to be involved in both structure and calcium handling in skeletal muscle, two characteristics of great importance for meat quality. The higher expression of *ANKRD1* in LW muscle associated with the great differences in meat quality between the 2 breeds, confirm the involvement of this gene in the biological processes determining pork quality, in agreement with Ponsuksili et al. [50] who suggested *ANKRD1* as a candidate gene for meat quality. Interestingly, *CASQ2* and *ATP2A1* (sarcoplasmic reticulum Ca^2+^-ATPase 1), a protein controlling the pumping of Ca^2+^ from the cytosol back to the sarcoplasmic reticulum, are both highly expressed in LW muscle. Besides, correlations between *ATP2A1* mutation and imf as well as muscle water content, suggest that *ATP2A1* locus could affect pork quality [51]. In conclusion, present results demonstrate the importance of muscle structure (cytoskeleton and sarcomere properties) in the differences found between breeds, thereby confirming the role of structural proteins in the determination of muscle and meat phenotypes [50] even though the relationships between gene expression and muscle traits remain further studies.

### Extracellular matrix

The LM of LW pigs is characterized by enrichment clusters of the cellular component GO terms for the extracellular region (cluster 1-LW) and the extracellular matrix part (clusters 2-LW and 3-LW). These clusters (representing 81 genes) exhibit the highest enrichment scores and statistical significance in this study, thus revealing their biological importance. *SPARC* and *SMOC2* (SPARC related modular calcium binding 2), two genes of these clusters, are members of the BM40 family which plays a key role in the cell-matrix interactions by promoting matrix assembly and cell adhesiveness. Especially, *SPARC* is a key matricellular protein involved in collagen I deposition and fibrillogenesis [29]. Since the LW exhibited higher muscle collagen content than the B pigs, *SPARC* gene expression could mediate this discrepancy. In this cluster, *DCN* (decorin) is involved in matrix assembly, and its targeted ablated expression strongly affects the collagen network [52]. *DCN* is also known to interact with *TGFB1* in satellite cells proliferation and differentiation [53]. Interestingly, *TGFB* receptor was found in the same cluster, suggesting that this interaction could explain LW muscle development. *DCN*, dermatopontin (*DPT*) and dystonin (*DST*) act in the same way since *DPT* accelerates the assembly of collagen into fibrils [54], whereas *DST* deficient mice exhibited weak skeletal muscle cytoarchitecture [55]. Moreover, six genes encoding various collagen types are highly expressed in LW muscle, in agreement with their higher collagen content. Thus, all these biological processes are in accordance with the LM properties of the LW compared with the B pigs, namely their elevated collagen content and shear force value, and lower tenderness score [56].

### Vacuole, lysosome and proteolysis

The vacuole and lysosome cluster (cluster 2-B) is a highly enriched CC cluster in the B pigs. Lysosomes contain many hydrolytic enzymes involved in the degradation of cytoplasmic proteins, even if the calpains and proteasome represent the main myofibibrillar proteolysis pathways [57]. Cathepsin D (*CTSD*, lysosomal protein) is highly expressed in the B pigs, and a mutation in *CSTD* gene has been associated with increased average daily gain and muscle mass, and decreased backfat deposition in both Duroc and LW pigs [58]. In this cluster, we also found *ATP6V1D* (ATPase, H+ transporting lysosomal), a subunit of a vacuole ATPase pumping protons from the cytoplasm to the lumen of the lysosome which might control pH homeostasis in muscle cells [59]. The potential role of *NEU1* (sialidase 1) in the control of cell proliferation, collagen content and extracellular matrix remodelling in skeletal muscle [60] and in inhibition of early myogenesis [61], agrees with the lower expression of *NEU1* found in LW pigs. This is also in accordance with their higher expression of collagen encoding genes and muscle development, and might thus be related to their lower meat tenderness.

The proteolysis function is not put forward by enrichment cluster analyses, but is at upmost importance in the context of meat tenderness. *CAST* (calpastatin), an inhibitor of calpain, one of the main proteolytic enzymatic systems involved in *post-mortem* muscle proteolysis and tenderization [62], was found highly expressed in LW breed. This could explain the higher shear force and lower tenderness score of the LW, as a result of a lower proteolysis level. Indeed, *CAST* has been suggested as a candidate gene for meat tenderness in pigs [63]. The ubiquitin-proteasome system, the main actor of nonlysosomal cytoplasmic protein degradation, appears to be also involved in the discrepancy between B and LW breeds with two out of the three ligase enzymes of the proteasome complex, *TRIM63* and *FBXO32* (F-box protein 32), more expressed in the B pigs. This could also contribute to the more tender meat of the B pigs, since proteasome would be one of the main endogenous proteolytic systems contributing to *post-mortem* meat tenderization [64].

In conclusion, our study aimed at identifying the biological events underlying differences in muscle physiology and meat quality traits reported in literature between the contrasted B and LW breeds. From transcriptomics and functional analyses, four main biological clusters were identified. Energy metabolism and lipid deposition are associated with breed-differences in muscle gene expressions and chemical composition. Furthermore, the cytoskeleton and the contractile fibers would play a role in the determination of muscle and meat phenotypes. Last, our results suggest the extracellular matrix as an important component of the LW muscle in accordance with their elevated collagen content, which could explain their reduced tenderness.

As a whole, our results contribute to a better understanding of muscle physiology and its consequences on the development of meat quality. Besides, this study is a first step towards the identification of molecular markers of pork quality and the subsequent development of control tools.

## Methods

### Ethics Statement

The experiment was conducted following French guidelines for animal care and use edited by the French Ministries of High Education and Research, and of Agriculture and Fisheries (http://ethique.ipbs.fr/sdv/charteexpeanimale.pdf). All animals were reared and slaughtered in compliance with national regulations and according to procedures approved by the French veterinary Services at INRA PEGASE facilities. Our research unit was holder of a pig experimentation agreement (N° A35622) delivered by the Veterinary Services of the French Ministry of Agriculture. Moreover, the technical and scientific staff involved in the experiment was holder of an individual agreement for experimentation on living animals delivered by the French Veterinary Services.

### Animals, husbandry and slaughtering

Forty finishing castrated males from a commercial selected LW pure breed (n = 20 issued from 10 litters produced from 9 different boars) pigs and an autochthonous B breed (n = 20 issued from 10 litters produced from 6 different boars) were reared in two different housing systems. At the average live weight of 35 kg, 2 pigs from each litter were chosen on the basis of their live weight and growth rate from birth up to 35 kg, and assigned to either a conventional (C), indoors on slatted floor (1.0 m^2^/pig), or an alternative (A) with indoor bedding (1.3 m^2^/pig) and a free access to an outdoor area (1.1 m^2^/pig) housing system at INRA-UMR PEGASE experimental farm, thus giving 4 groups of 10 pigs per breed and housing system. Pigs of both housing systems were fed a standard commercial diet with 2.5 kg/d/pig from 35 up to 110 kg, and 3.0 kg/d/pig up to slaughter at the average weight of 145 kg. Pigs were slaughtered at INRA-UMR PEGASE experimental slaughterhouse in four sessions (each including pigs from each breed and housing system), by electrical stunning and exsanguination.

### Carcass, muscle and meat quality measurements

The day of slaughter, hot carcass weight and back fat thickness (mid line, between 4^th^ and 5^th^ lumbar vertebra level) were recorded. After 24 h at 4°C, the fresh carcass and wholesale cuts of right side were weighted for calculation of loin and backfat proportions. Thirty minutes after exsanguination, a sample of LM was carefully collected on all pigs (right half-carcass, last rib level) immediately frozen in liquid nitrogen and stored at −80°C until RNA extraction (see below) and determination of glycolytic potential [13]. The following day, a transversal slice of LM was taken (1^st^ lumbar vertebra level), trimmed of external fat and minced. Half of this sample was put under vacuum and stored at −20°C until determination of intramuscular fat content, and the remaining minced LM was freeze-dried before determination of protein and collagen contents, as previously described [65]. Water content was determined from the weight of minced muscle before and after freeze-drying, and used for calculations of protein and collagen content per gram of fresh muscle. The day after slaughter, another slice (1.5 cm depth) was taken of the LM (the 2^nd^ vertebra level), weighed (100±10 g) and kept for 48 h at 4°C in plastic bags for determination of drip loss [66]. On all pigs, a piece of the left loin (between the 2^nd^/3^rd^ and 6^th^/7^th^ last ribs) was taken 24 h after slaughter, partially trimmed of external fat, kept at 4°C for 3 subsequent days, and deboned. The LM was put under vacuum, frozen and stored at −20°C before determination of shear force on cooked meat using a Warner-Bratzler cell fitted on a universal testing machine (Instron France S.A.S., Guyancourt, France) according to Honikel [66]. The shear force mean values were obtained from 10 measurements per LM sample. A piece of the right loin (between the 2^nd^/3^rd^ and 9^th^/10^th^ last ribs) was also taken on all pigs the day after slaughter, prepared and stored as described above until sensory analysis performed at INRA-EASM (Le Magneraud, Surgères, France). The 40 roasts were evaluated over 10 sessions, each including four roasts, one per breed and per rearing system. After thawing for 48 h at 4°C, roasts (900 g) were cooked in an oven (dry heat, 250°C for 10 min, followed by humid heat, 100°C for around 45 min up to a core temperature of 80±2°C). Then, the middle part of 1-cm thick slices of roasts was presented to the 12 panellists who evaluated tenderness, juiciness and flavour on a continuous scale form 0 (absent) to 10 (very high intensity of the trait). The average of individual panellist scores from each sample was used for the statistical analysis.

Carcass, muscle and meat quality data were submitted to an analysis of variance (GLM procedure, SAS Inst. Inc., Cary, NC). The model included the fixed effects of breed, housing system, and their interaction. Least square (LS) means were calculated per breed and per housing system.

### Microarray design

A custom pig skeletal muscle microarray [14] of 15198 oligonucleotides (60 mers) was used in this study. Among the 15198 probes of the GenmascqChip, 12939 probes (i.e. 85% of the oligonucleotides) have been linked to a unique annotated sequence and to 9169 unique genes (i.e., 30% of redundancy). An 8×15 K oligo-microarray Agilent format was chosen, therefore one probe per microarray and eight microarrays were fitted in each slide.

### RNA extraction and Microarray hybridization

Total RNA was extracted by crushing the frozen tissue in Trizol reagent (Invitrogen, Cergy-Pontoise, France) and purification using a silica-based spin-column (RNA II kit, Macherey Nagel, Lyon, France). The quality and concentration of total RNA were verified by electrophoresis using an Agilent Analyzer (Agilent Technologies France, Massy, France) and UV spectrometry (Nanodrop, Thermo Scientific, Illkirch, France). In order to compare the 40 LM samples among the experiment, each sample was compared to a reference pool composed of an equal amount of transcripts isolated from all 40 LM samples. Total RNA (350 ng) from each animal was labeled individually with Cy3 and the reference sample was labeled with Cy5, using the Quick-Amp Labeling Kit (Agilent Technologies, Santa Clara, USA) and following the manufacturer's instructions. Microarray hybridizations were carried out in Agilent's SureHyb Hybridization Chambers containing 300 ng of Cy3-labeled cRNA sample and 300 ng of Cy5-labeled reference sample per hybridization. The hybridization reactions were performed at 65°C for 17 hours using Agilent's Gene Expression Hybridization Kit. Slides were disassembled and washed in Gene Expression Wash Buffer 1 for 1 minute at room temperature and then in Gene Expression Wash Buffer 2 for 1 minute. Microarrays were scanned at 5 µm/pixel resolution using the Agilent DNA Microarray Scanner G2505B, and images were analyzed with Agilent Feature Extraction Software (Version 9.5), using the GE2-v5_95_Feb07 FE extraction protocol. These MIAME compliant microarray data have been deposited into the GEO [15] repository and are publicly available through GEO Series accession no. GSE26614.

### Microarray Data Analyses and Statistics

All analyses were performed using the R software version 2.8.1 [67]. Raw spots intensities were first submitted to quality filtration based on four criteria: intensity, uniformity, saturation and outliers detection. Intensities of filtered spots were transformed into log_2_ (Cy3/Cy5). Data were normalized within chips by subtraction of the sample median value across all probes from all raw values, and between chips using the “Rquantile” method of the Limma R package [68] to obtain experimentally consolidated gene expression values. The “Rquantile” method was used since the red channel was the common reference throughout the experiment. To increase the power of differential expression analysis [69], spots with the smallest expression variability across samples were filtered out using K-means algorithm (k = 4). All together, 4870 spots were finally retained for statistical analyses. Expression data were then adjusted for slide effect when significant (p<0.05) by analysis of variance, before performing differential expression analysis. Residuals were then submitted to an analysis of variance using the fixed effects of breed (B or LW), housing system (C or A) and their interaction (breed×housing system). Data were then submitted to Benjamini and Hochberg (BH) multiple testing correction procedure [70] using an adjusted P value cutoff of 0.05.

### Functional analysis

Enrichment analysis for specific GO terms for BP has been carried out using the DAVID [17]–[19]. In DAVID analysis, the GO _FAT terms were selected to filter the broadest terms without overshadow the more specific ones. The lists of genes were uploaded using the ENTREZ gene ID (http://www.ncbi.nlm.nih.gov/gene). The P values for enrichment (or EASE scores) were computed by a modified Fisher's exact test, using our custom microarray (i.e. 8639 human ENTREZ gene ID) as background. The GO categories (BP, CC and MF) and KEGG and Biocarta pathways were clustered using the DAVID Functional Annotation Clustering tool [17]–[19], where the enrichment score for each cluster was computed as the negative log of the geometric mean of P values in the cluster.

### Real Time PCR analysis

Complementary DNA was synthesised from 2 µg of total RNA previously used for microarray analysis, using the High Capacity cDNA Reverse Transcription Kit (Applied Biosystems, Foster City, CA). Primers were designed using Primer Express Software (Applied Biosystems, USA) based on *Sus scrofa* published nucleotides sequences (Table S3). Amplification was performed in triplicate, in 12.5 µl with 2.5 ng of reverse-transcribed RNA and both forward and reverse primers (200 nM each) in 1× PCR buffer (Fast SYBR® Green Master Mix, Applied Biosystems) containing Uracil DNA glycosylase to prevent any DNA contamination from previous PCR. A StepOnePlus™ Real Time PCR system (Applied Biosystems) was used. Thermal cycling conditions were as follows: 50°C for 2 min, 95°C for 20 s, followed by 40 cycles of denaturation at 95°C for 3 s, and annealing at 60°C for 30 s. Specificity of the amplification products was checked by dissociation curves analysis. Three genes were used as reference for normalization: *HPRT1* (hypoxanthine phosphoribosyltransferase 1), *B2M* (beta-2 microglobulin) and *18S* (18S rRNA predeveloped TaqMan kit from Applied Biosystems). Using geNorm [71] and Normfinder [72] algorithms, all three genes appeared to have a stable expression on all LM samples. For each sample, the normalized expression level (N_exp_) was calculated according to the following formula: N_exp_ = (1+E)^−ΔCt (sample−calibrator)^/NF, where the calibrator is a pool of the 40 LM samples, E is the PCR efficiency and NF is the normalization factor calculated using geNorm algorithm. Normalized expression levels of mRNAs were then compared between B and LW samples using the Student t-test and P value≤0.05 for significance.

## Supporting Information

Table S1 **Genes highly expressed in Basque pigs.** Results were expressed as the Basque to Large White ratio of the gene expression. The p value of each gene was adjusted according to the Benjamini-Hochberg method. Difference in gene expression was considered significant if its adjusted p value was p<0.05. Redundancy represented the number of probes per gene. In this list, 73 genes had more than one probe.
(XLS)

Table S2 **Genes highly expressed in Large White pigs.** Results were expressed as the Basque to Large White ratio of the gene expression. The p value of each gene was adjusted according to the Benjamini-Hochberg method. Difference in gene expression was considered significant if its adjusted p value was p<0.05. Redundancy represented the number of probes per gene. In this list, 75 genes had more than one probe.
(XLS)

Table S3 **Primer sequences used in quantitative PCR.** All primer sequences were designed using PrimerExpress software (Applied Biosystems, Foster City, CA). *^a^HPRT1* and *B2M* were used as reference for normalization.
(XLS)

## References

[1] Stewart TS, Lofgren DL, Harris DL, Einstein ME, Schinckel AP (1991). Genetic improvement programs in livestock: swine testing and genetic evaluation system (stages).. J Anim Sci.

[2] Tribout T, Caritez JC, Gruand J, Bouffaud M, Guillouet P (2010). Estimation of genetic trends in French Large White pigs from 1977 to 1998 for growth and carcass traits using frozen semen.. J Anim Sci.

[3] Schwab CR, Baas TJ, Stalder KJ, Mabry JW (2006). Effect of long-term selection for increased leanness on meat and eating quality traits in Duroc swine.. J Anim Sci.

[4] Rosenvold K, Andersen HJ (2003). Factors of significance for pork quality – a review.. Meat Sci.

[5] Laval G, Iannuccelli N, Legault C, Milan D, Groenen MA (2000). Genetic diversity of eleven European pig breeds.. Genet Sel Evol.

[6] Alfonso L, Mourot J, Insaustia K, Mendizabala JA, Arana A (2005). Comparative description of growth, fat deposition, carcass and meat quality characteristics of B and LW pigs.. Anim Res.

[7] Cánovas A, Quintanilla R, Amills M, Pena RN (2010). Muscle transcriptomic profiles in pigs with divergent phenotypes for fatness traits.. BMC Genomics.

[8] Ponsuksili S, Murani E, Phatsara C, Jonas E, Walz C (2008). Expression profiling of muscle reveals transcripts differentially expressed in muscle that affect water-holding capacity of pork.. J Agric Food Chem.

[9] Wimmers K, Murani E, Ponsuksili S (2010). Pre-and postnatal differential gene expression with relevance for meat and carcass traits in pigs – a review.. Anim Sci Pap Rep.

[10] Zhao X, Mo D, Li A, Gong W, Xiao S (2011). Comparative analyses by sequencing of transcriptomes during skeletal muscle development between pig breeds differing in muscle growth rate and fatness.. PLos One.

[11] Davoli R, Braglia S (2007). Molecular approaches in pig breeding to improve meat quality.. Brief Funct Genomic Proteomic.

[12] Labroue F, Goumy S, Gruand J, Mourot J, Neelz V (2000). Comparaison au Large White de 4 races locales porcines françaises pour les performances de croissance, de carcasse et de qualité de la viande.. Journées Rech Porcine en France.

[13] Lebret B, Meunier-Salaün MC, Foury A, Mormède P, Dransfield E (2006). Influence of rearing conditions on performance, behavioral, and physiological responses of pigs to preslaughter handling, carcass traits, and meat quality.. J Anim Sci.

[14] Damon M, Herault F, Vincent A, Le Roy P, Cherel P (2011). Characterization of a pig skeletal muscle microarray to study pork quality: the GENMASCQ Chip 15 K.. Nat Prec.

[15] Gene Expression Omnibus (GEO).. http://www.ncbi.nlm.nih.gov/geo/.

[16] WEB-based-Gene-SeT-AnaLysis-Toolkit.. http://bioinfo.vanderbilt.edu/webgestalt/.

[17] Database for Annotation, Visualization and Integrated Discovery.. http://david.abcc.ncifcrf.gov/home.jsp.

[18] Dennis G, Sherman BT, Hosack DA, Yang J, Gao W (2003). DAVID: Database for Annotation, Visualization, and Integrated Discovery.. Genome Biol.

[19] Huang DW, Sherman BT, Lempicki RA (2009). Systematic and integrative analysis of large gene lists using DAVID Bioinformatics Resources.. Nature Protoc.

[20] Kanehisa M, Goto S (2000). KEGG: kyoto encyclopedia of genes and genomes.. Nucleic Acids Res.

[21] Kim NK, Park HR, Lee HC, Yoon D, Son ES (2010). Comparative studies of skeletal muscle proteome and transcriptome profilings between pig breeds.. Mamm Genome.

[22] Lin CS, Hsu CW (2005). Differentially transcribed genes in skeletal muscle of Duroc and Taoyuan pigs.. J Anim Sci.

[23] Lebret B (2008). Effects of feeding and rearing systems on growth, carcass composition and meat quality in pigs.. Animal.

[24] Bonneau M, Lebret B (2010). Production systems and influence on eating quality of pork.. Meat Sci.

[25] Gerbens F, Verburg FJ, Van Moerkerk HT, Engel B, Buist W (2001). Associations of heart and adipocyte fatty acid-binding protein gene expression with intramuscular fat content in pigs.. J Anim Sci.

[26] Arnyasi M, Grindflek E, Jávor A, Lien S (2006). Investigation of two candidate genes for meat quality traits in a quantitative trait locus region on SSC6: the porcine short heterodimer partner and heart fatty acid binding protein genes.. J Anim Breed Genet.

[27] Tomsig JL, Snyder AH, Berdyshev EV, Skobeleva A, Mataya C (2009). Lipid phosphate phosphohydrolase type 1 (LPP1) degrades extracellular lysophosphatidic acid in vivo.. Biochem J.

[28] Shan T, Wu T, Reng Y, Wang Y (2009). Breed difference and regulation of the porcine adipose triglyceride lipase and hormone sensitive lipase by TNFalpha.. Anim Genet.

[29] Bradshaw AD (2009). The role of SPARC in extracellular matrix assembly.. J Cell Commun Signal.

[30] Bao Y, Dawson TL, Chen YT (1996). Human glycogen debranching enzyme gene (AGL): complete structural organization and characterization of the 5′ flanking region.. Genomics.

[31] Wüllrich-Schmoll A, Kilimann MW (1996). Structure of the human gene encoding the phosphorylase kinase beta subunit (PHKB).. Eur J Biochem.

[32] Lefaucheur L (2010). A second look into myofiber typing – relation to meat quality.. Meat Sci.

[33] van de Wiel DFM, Zhang WL (2007). Identification of pork quality parameters by proteomics.. Meat Sci.

[34] Sayd T, Morzel M, Chambon C, Franck M, Figwer P (2006). Proteome analysis of the sarcoplasmic fraction of pig semimembranosus muscle: implications on meat color development.. J Agric Food Chem.

[35] Schmidt A, Hall MN (1998). Signaling to the actin cytoskeleton.. Annu Rev Cell Dev Biol.

[36] Kuwahara K, Barrientos T, Pipes GC, Li S, Olson EN (2005). Muscle-specific signaling mechanism that links actin dynamics to serum response factor.. Mol Cell Biol.

[37] Lamon S, Wallace MA, Léger B, Russell AP (2009). Regulation of STARS and its downstream targets suggest a novel pathway involved in human skeletal muscle hypertrophy and atrophy.. J Physiol.

[38] Barrientos T, Frank D, Kuwahara K, Bezprozvannaya S, Pipes GC (2007). Two novel members of the ABLIM protein family, ABLIM-2 and -3, associate with STARS and directly bind F-actin.. J Biol Chem.

[39] Tsukada T, Pappas CT, Moroz N, Antin PB, Kostyukova AS (2010). Leiomodin-2 is an antagonist of tropomodulin-1 at the pointed end of the thin filaments in cardiac muscle.. J Cell Sci.

[40] Wheeler TL, Shackelford SD, Koohmaraie M (2000). Variation in proteolysis, sarcomere length, collagen content, and tenderness among major pork muscles.. J Anim Sci.

[41] Honikel KO, Kim CJ, Hamm R, Roncales P (1986). Sarcomere shortening of prerigor muscles and its influence on drip loss.. Meat Sci.

[42] Frey N, Frank D, Lippl S, Kuhn C, Kögler H (2008). Calsarcin-2 deficiency increases exercise capacity in mice through calcineurin/NFAT activation.. J Clin Invest.

[43] Sakuma K, Yamaguchi A (2010). The functional role of calcineurin in hypertrophy, regeneration, and disorders of skeletal muscle.. J Biomed Biotechnol.

[44] Bodine SC, Latres E, Baumhueter S, Lai VK, Nunez L (2001). Identification of ubiquitin ligases required for skeletal muscle atrophy.. Science.

[45] Smith MA, Blankman E, Gardel ML, Luettjohann L, Waterman CM (2010). A zyxin-mediated mechanism for actin stress fiber maintenance and repair.. Dev Cell.

[46] Labeit S, Ottenheijm CA, Granzier H (2011). Nebulin, a major player in muscle health and disease.. FASEB J.

[47] Lee SH, Cho YM, Lee SH, Kim BS, Kim NK (2008). Identification of marbling-related candidate genes in M. longissimus dorsi of high- and low marbled Hanwoo (Korean Native Cattle) steers.. BMB Rep.

[48] Samaras SE, Shi Y, Davidson JM (2006). CARP: fishing for novel mechanisms of neovascularization.. J Investig Dermatol Symp Proc.

[49] Torrado M, Nespereira B, López E, Centeno A, Castro-Beiras A (2005). ANKRD1 specifically binds CASQ2 in heart extracts and both proteins are co-enriched in piglet cardiac Purkinje cells.. J Mol Cell Cardiol.

[50] Ponsuksili S, Murani E, Phatsara C, Schwerin M, Schellander K (2009). Porcine muscle sensory attributes associate with major changes in gene networks involving CAPZB, ANKRD1, and CTBP2.. Funct Integr Genomics.

[51] Chai J, Xiong Q, Zhang PP, Shang YY, Zheng R (2010). Evidence for a new allele at the SERCA1 locus affecting pork meat quality in part through the imbalance of Ca2+ homeostasis.. Mol Biol Rep.

[52] Danielson KG, Baribault H, Holmes DF, Graham H, Kadler KE (1997). Targeted disruption of decorin leads to abnormal collagen fibril morphology and skin fragility.. J Cell Biol.

[53] Li X, McFarland DC, Velleman SG (2008). Extracellular matrix proteoglycan decorin-mediated myogenic satellite cell responsiveness to transforming growth factor-beta1 during cell proliferation and differentiation: Decorin and transforming growth factor-beta1 in satellite cells.. Domest Anim Endocrinol.

[54] MacBeath JR, Shackleton DR, Hulmes DJ (1993). Tyrosine-rich acidic matrix protein (TRAMP) accelerates collagen fibril formation in vitro.. J Biol Chem.

[55] Dalpé G, Mathieu M, Comtois A, Zhu E, Wasiak S (1999). Dystonin-deficient mice exhibit an intrinsic muscle weakness and an instability of skeletal muscle cytoarchitecture.. Dev Biol.

[56] Purslow PP (2004). Intramuscular connective tissue and its role in meat quality.. Meat Sci.

[57] Goll DE, Neti G, Mares SW, Thompson VF (2008). Myofibrillar protein turnover: the proteasome and the calpains.. J Anim Sci.

[58] Fontanesi L, Speroni C, Buttazzoni L, Scotti E, Dall'Olio S (2010). The insulin-like growth factor 2 (IGF2) gene intron3-g.3072G>A polymorphism is not the only Sus scrofa chromosome 2p mutation affecting meat production and carcass traits in pigs: evidence from the effects of a cathepsin D (CTSD) gene polymorphism.. J Anim Sci.

[59] Finbow ME, Harrison MA (1997). The vacuolar H+-ATPase: a universal proton pump of eukaryotes.. Biochem J.

[60] Zanoteli E, van de Vlekkert D, Bonten EJ, Hu H, Mann L (2010). Muscle degeneration in neuraminidase 1-deficient mice results from infiltration of the muscle fibers by expanded connective tissue.. Biochim Biophys Acta.

[61] Champigny MJ, Perry R, Rudnicki M, Igdoura SA (2005). Overexpression of MyoD-inducible lysosomal sialidase (neu1) inhibits myogenesis in C2C12 cells.. Exp Cell Res.

[62] Hopkins DL, Geesink GH, Du M, McCormick RJ (2009). Protein degradation post-mortem and tenderization.. Applied muscle biology and meat science.

[63] Meyers SN, Beever JE (2008). Investigating the genetic basis of pork tenderness: genomic analysis of porcine CAST.. Anim Genet.

[64] Sentandreu MA, Coulis G, Ouali A (2002). Role of muscle endopeptidases and their inhibitors in meat tenderness.. Trends Food Sci Techno.

[65] Lebret B, Heyer A, Gondret F, Louveau I (2007). The response of various muscle types to a restriction-realimentation feeding strategy in growing pigs.. Animal.

[66] Honikel KO (1998). Reference methods for the assessment of physical characteristics of meat.. Meat Sci.

[67] R Development Core Team (2008). R: A language and environment for statistical computing.. http://www.R-project.org.

[68] Smyth GK, Speed TP (2003). Normalization of cDNA microarray data.. Methods.

[69] Hackstadt AJ, Hess AM (2009). Filtering for increased power for microarray data analysis.. BMC Bioinformatics.

[70] Benjamini Y, Hochberg Y (1995). Controlling the false discovery rate, a practical and powerful approach to multiple testing.. J Royal Statist Soc Ser B.

[71] Vandesompele J, De Preter K, Pattyn F, Poppe B, Van Roy N (2002). Accurate normalization of real-time quantitative RT-PCR data by geometric averaging of multiple internal control genes.. Genome Biol.

[72] Andersen CL, Ledet-Jensen J, Ørntoft T (2004). Normalization of real-time quantitative RT-PCR data: a model based variance estimation approach to identify genes suited for normalization - applied to bladder- and colon-cancer data-sets.. Cancer Research.

